# Proinflammatory and Immunoregulatory Roles of Eicosanoids in T Cells

**DOI:** 10.3389/fimmu.2013.00130

**Published:** 2013-06-04

**Authors:** Anna Mari Lone, Kjetil Taskén

**Affiliations:** ^1^Centre for Molecular Medicine Norway, Nordic EMBL Partnership, University of Oslo and Oslo University Hospital, Oslo, Norway; ^2^Biotechnology Centre, University of Oslo, Oslo, Norway; ^3^K.G. Jebsen Inflammation Research Centre, University of Oslo, Oslo, Norway; ^4^Department of Infectious Diseases, Oslo University Hospital, Oslo, Norway

**Keywords:** prostaglandins, leukotrienes, cyclooxygenase 2, regulatory T cells, cAMP, immunoregulation effect, inflammation mediators, inflammation

## Abstract

Eicosanoids are inflammatory mediators primarily generated by hydrolysis of membrane phospholipids by phospholipase A2 to ω-3 and ω-6 C_20_ fatty acids that next are converted to leukotrienes (LTs), prostaglandins (PGs), prostacyclins (PCs), and thromboxanes (TXAs). The rate-limiting and tightly regulated lipoxygenases control synthesis of LTs while the equally well-controlled cyclooxygenases 1 and 2 generate prostanoids, including PGs, PCs, and TXAs. While many of the classical signs of inflammation such as redness, swelling, pain, and heat are caused by eicosanoid species with vasoactive, pyretic, and pain-inducing effects locally, some eicosanoids also regulate T cell functions. Here, we will review eicosanoid production in T cell subsets and the inflammatory and immunoregulatory functions of LTs, PGs, PCs, and TXAs in T cells.

## Introduction

The eicosanoids constitute a large and expanding family of lipid signaling molecules derived from ω-3 and ω-6 C_20_ fatty acids (Smith, 1989; Funk, 2001). This conversion of membrane phospholipids into potent signaling mediators provides an efficient way for cells to respond to various stimuli that require a cellular response. As part of a complex network of regulators controlling a number of important physiological properties including smooth muscle tone, vascular permeability, and platelet aggregation, eicosanoids have also been implicated in a wide array of pathophysiological processes and diseases, including inflammation, autoimmunity, allergy, HIV, and cancer (Harizi et al., 2008; Greene et al., 2011; Bertin et al., 2012). While eicosanoids, in particular prostaglandins, were originally thought of primarily as proinflammatory mediators given their high expression in inflamed tissues and ability to induce inflammatory symptoms, this picture has over time become more nuanced. It is now recognized that these lipids can have both pro- and anti-inflammatory roles by regulating the immune response (Tilley et al., 2001).

**Figure 1:**
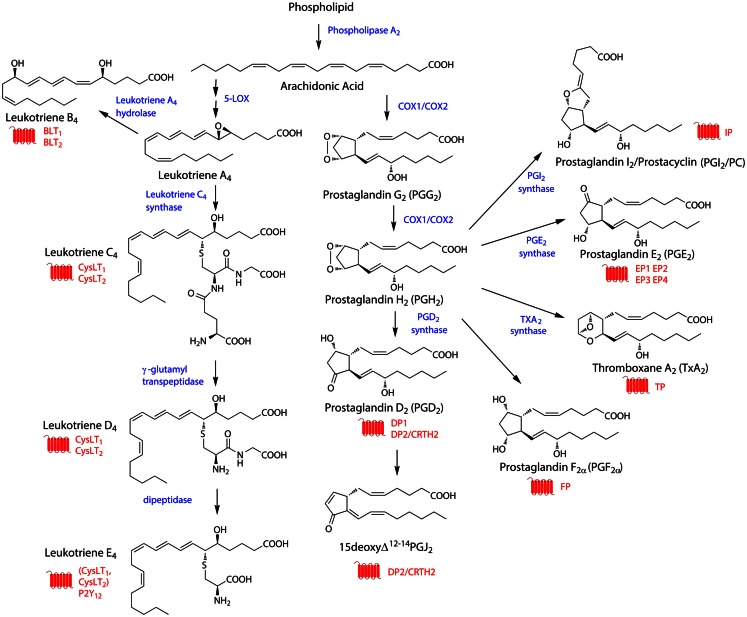
**General overview of synthesis pathways for eicosanoids**. The biosynthetic pathway for the arachidonic acid-derived eicosanoids described in this article. The figure shows the structures of the relevant eicosanoids (black), and indicates the enzymes involved in their biosynthesis (blue), as well as the GPCRs through which these eicosanoids signal (red). PGF_2α_ can be synthesized through a number of different pathways.

While some eicosanoids are produced from eicosapentaenoic acid (EPA, 20:5 ω-3) (Wada et al., 2007) or dihomo-γ-linolenic acid (DGLA, 20:3 ω-6), the majority arise from processing of arachidonic acid (AA, 20:4 ω-6) (Harizi et al., 2008). AA-derived eicosanoids comprise the P-450 epoxygenase-generated hydroxyeicosatetraenoic acids (HETEs) and epoxides, the lipoxygenase (LOX) – generated hydroperoxyeicosatetraenoic acids (HPETEs), lipoxins (LXs), and leukotrienes (LTs), and the cyclooxygenase (COX)-produced prostanoids (see Figure 1 for overview of biosynthetic pathways). The prostanoids are perhaps the most well-known class of eicosanoids and include the prostaglandins (PGs) PGD_2_, PGE_2_, and PGF_2α_ as well as prostacyclin (PC/PGI_2_) and thromboxane (TXA_2_). Together with the leukotrienes, the AA-derived prostanoids will be the major focus of this article.

Constitutive eicosanoid production is normally low, with the rate-limiting factor being the availability of free fatty acids, in particular AA. Free fatty acids are generated from membrane glycerophospholipids by phospholipase A_2_s (PLA_2_s) (Kudo and Murakami, 2002; Leslie, 2004; Burke and Dennis, 2009) in response to stimuli such as increased Ca_2+_ levels or phosphorylation (Kudo and Murakami, 2002). This elevation in intracellular free fatty acid levels, in particular that of arachidonic acid, then allows eicosanoid biosynthesis to proceed. In the case of prostanoid biosynthesis, AA is converted into PGG_2_ and then PGH_2_ through the actions of COX-1 and COX-2 (also known as PGH synthases 1 and 2). These enzymes act first as cyclooxygenases to create PGG_2_ and then as peroxidases to reduce the peroxide in PGG_2_ to an alcohol in PGH_2_ (Smith et al., 2000, 2011). Both PGG_2_ and PGH_2_ are thought to be transient intermediates and their production constitutes the committed step in prostanoid biosynthesis. PGH_2_ is then converted into one of four possible downstream signaling molecules (Figure 1). Prostacyclin synthase (PGIS) converts PGH_2_ to PGI_2_, hematopoietic (H-PGDS) or lipocalin-type (L-PGDS) PGD_2_ synthase convert PGH_2_ into PGD_2_, TXA_2_ synthase (TXAS) converts PGH_2_ into TXA_2_, and membrane-bound (mPGES-1 or -2) or cytosolic (cPGES) PGE_2_ synthases convert PGH_2_ into PGE_2_. PGF_2α_ can be synthesized through a number of different pathways (Basu, 2010; Smith et al., 2011).

On the other hand, in leukotriene biosynthesis, AA is not processed by COX enzymes, but instead by 5-LOX, which with the help of 5-LOX -activating protein (FLAP) converts AA first into 5-HPETE and then into LTA_4_, an inactive intermediate and precursor for other leukotrienes. LTA_4_ can either be converted into LTB_4_ by LTA_4_ hydrolase or into LTC_4_ by LTC_4_ synthase, which conjugates a glutathione to LTA_4_ (Yokomizo, 2011). LTC_4_ can then be converted sequentially to LTD_4_ by gamma-glutamyl transpeptidase and LTE_4_ by dipeptidases (Brink et al., 2003).

By signaling through their receptors on the surface of T cells, eicosanoids have an important role in regulating many aspects of T lymphocyte function, usually through autocrine or paracrine signaling (Tilley et al., 2001; Sakata et al., 2010a). It has also recently become evident that T cells provide a source of these short-lived signaling mediators in compartments such as lymph nodes and spleen and in lymphoid infiltrates. In the present review, we will summarize the evidence for the production of and signaling by these molecules in T cells, especially in the context of the regulation of immunomodulatory or inflammatory functions.

## Biosynthesis of and Signaling by Eicosanoids in T Lymphocytes

### PGG_2_ and PGH_2_

Production of PGG_2_ and PGH_2_ proceeds through the actions of PLA_2_ and COX-1 or COX-2. While there has been some discussion about which PLA_2_ variant(s) are most relevant for eicosanoid biosynthesis, it is generally agreed that cytosolic PLA_2α_ (cPLA_2α_) plays a major role in this process, with the Ca_2+_-independent PLA_2_ (iPLA_2_) more involved in membrane homeostasis and secreted PLA_2_ (sPLA_2_) regulating extracellular phospholipids (Murakami et al., 2011). Expression of cPLA_2α_ in T cells has been controversial, with some groups finding no evidence for it in peripheral blood monocytes (Roshak et al., 2000) while others have observed mRNA but no effect of inhibiting this enzyme on AA production in Jurkat T cells (Tessier et al., 2002) and yet others observe this protein both in Jurkat T cells and peripheral blood lymphocytes (Burgermeister et al., 2003) (see Figure 1 for overview of biosynthetic enzymes and Table 1 for their expression in T cells).

**Table 1:** **Eicosanoid synthesis in T cells**.

Synthase	Presence in T cells
PLA_2_	cPLA_2α_: Jurkat (Tessier et al., 2002; Burgermeister et al., 2003)
	iPLA_2_: Jurkat, primary T cells (Roshak et al., 2000; Tessier et al., 2002)
	sPLA_2_: Jurkat (Tessier et al., 2002)
COX-1	CD3+CD4+ primary T cells, Jurkat (Iniguez et al., 1999; Pablos et al., 1999)
COX-2	CD3+CD4+ primary T cells, Jurkat (Iniguez et al., 1999; Pablos et al., 1999). Upregulated upon T cell activation (Feldon et al., 2006). Expressed also in adaptive Tregs (Mahic et al., 2006)
PGIS	Lymphocytes (Merhi-Soussi et al., 2000). No specific evidence for expression in T cells
PGDS	L-PGDS: not present in T cells
	H-PGDS: present in primary T cells (Feldon et al., 2006), in particular activated Th2 and Tc2 cells (Tanaka et al., 2000)
TXAS	No direct evidence for expression in T cells. However, the presence of TXAS products in some T cells indicates that it may be expressed at low levels (Genaro et al., 1992; Kabashima et al., 2003)
PGES	No direct evidence, but product is present in Tregs, implying expression (Mahic et al., 2006)
PGFS	No evidence for expression in T cells
5-LOX	Present in peripheral blood T cells, including naive and memory CD4+ and CD8+ as well as TCR–γδ cells (Cook-Moreau et al., 2007). Also T cell lines (Cook-Moreau et al., 2007)
LTC4S	Jurkat (Cook-Moreau et al., 2007), peripheral blood T cells (Cifone et al., 1995)
LTA4H	Jurkat (Cook-Moreau et al., 2007), peripheral blood T cells (Los et al., 1995)

Some groups have concluded that other PLA_2_ variants may also be active in T cells, with evidence for both iPLA_2_ (Roshak et al., 2000; Tessier et al., 2002) and sPLA_2_ (Tessier et al., 2002) being present and active in T lymphocytes. Interestingly, an sPLA_2_ isoform has also been shown to be expressed in and enhance the function of regulatory T cells (Treg), but this effect was found to be independent of the enzyme’s catalytic activity (von Allmen et al., 2009).

Alternatively, arachidonic acid can be released from membrane phospholipids by phospholipase D (PLD) (Liscovitch et al., 2000; Melendez and Allen, 2002), which has been shown to be inducible in human T cells (Bacon et al., 1995, 1998; Exton, 1999). Diacylglycerol (DAG) lipase has also been shown to play a role in the release of AA in lymphocytes (Cifone et al., 1995).

COX-1 and COX-2, which are capable of converting AA into PGH_2_, are both expressed in CD3+CD4+ cells and in Jurkat T cells. COX-1 is expressed constitutively in T cells and does not change in response to T cell activation (Pablos et al., 1999). In contrast, COX-2 is normally expressed at low levels but significantly upregulated in response to T cell activation (Iniguez et al., 1999; Pablos et al., 1999; Feldon et al., 2006). A study from this lab further demonstrated that during differentiation of adaptive Tregs, these cells also begin expressing COX-2 and producing PGE_2_ (Mahic et al., 2006).

In the context of a discussion of the cellular localizations of PLA_2_ and COX enzymes, it is worth noting that transcellular eicosanoid biosynthesis has recently been proposed as a mechanism whereby the entire biosynthetic pathway for a given eicosanoid need not be present in one particular cell. Instead, the synthesis may begin in one cell, followed by the transfer of a synthetic intermediate to a different cell where the final product is synthesized. PGH_2_, LTA_4_, and arachidonic acid have all been proposed as possible intermediates transported between cells, suggesting that in some cases, PLA_2_ (and COX enzymes) could be present in one cell and the remaining synthases required for prostanoid or leukotriene synthesis in another (Folco and Murphy, 2006; Sala et al., 2010). For PGH_2_, it has also been proposed that two distinct pathways for PGH_2_ synthesis exist: one for production of PGH_2_ to be converted into downstream prostanoids in the usual manner and one for production of untransformed PGH_2_ to be released for signaling functions.

PGG_2_ is a transient intermediate in prostanoid biosynthesis, with a half-life of about 5 min in aqueous solution at 37 °C, pH 7.4 and significantly shorter – on the order of seconds – in plasma (Corey et al., 1975). Although there have been some suggestions that PGG_2_ may have a biological function (Kuehl et al., 1977; Seidel et al., 2001) it is primarily considered an ephemeral intermediate without independent signaling functions. There is no evidence for a signaling role of this species in T cells.

In the case of PGH_2_, this is also an unstable endoperoxide species with comparable half-life to that of PGG_2_ (Corey et al., 1975). Although no specific receptor has been identified for this species either, there is some evidence that it can interact with other prostanoid receptors, including the DP and CRTH2 receptors (Schuligoi et al., 2009) as well as the TP receptor (Saito et al., 2003). Because of the rapid conversion of both PGG_2_ and PGH_2_ to other prostanoid species, however, it has been challenging to unequivocally prove that there is a direct action of these intermediate species on any of the prostanoid receptors. Several of the receptors thought to be activated by PGH_2_, in particular CRTH2, are known to be expressed on T cells, but so far it has not been demonstrated that PGH_2_ has a biologically relevant role in activating these receptors when expressed on T cells *in vivo* (See Figure 1 for overview of receptors and Table 2 for overview of expression of eicosanoid receptors in T cells).

**Table 2:** **Eicosanoid receptors in T cells**.

Receptor	Present in which T cells
IP	T lymphocytes (Tilley et al., 2001), in particular Th1 and Th2 (Zhou et al., 2007)
TP	T lymphocytes (Tilley et al., 2001). Highly expressed in immature thymocytes (CD4+CD8+ and CD4−CD8−) and present in mature CD4+ and CD8+ cells (Ushikubi et al., 1993; Kabashima et al., 2003) and in splenic T cells (Ushikubi et al., 1993)
DP1	Th1, Th2, and CD8+ (Tanaka et al., 2004), CD3+ cells in thymus and lymph nodes (Nantel et al., 2004)
DP2/CRTH2	T lymphocytes (Tilley et al., 2001), activated Th2 and Tc2 cells (Hirai et al., 2001; Tsuda et al., 2001; Tanaka et al., 2004)
EP1	T lymphocytes (Tilley et al., 2001), splenic T cells (Nataraj et al., 2001), low expression in peripheral blood naive T cells (Boniface et al., 2009)
EP2	T lymphocytes (Tilley et al., 2001), splenic T cells (Nataraj et al., 2001), peripheral blood naive T cells, upregulated upon T cell activation (Boniface et al., 2009)
EP3	T lymphocytes (Tilley et al., 2001), splenic T cells (not α, β isoforms) (Nataraj et al., 2001), low expression in peripheral blood naive T cells (Boniface et al., 2009)
EP4	T lymphocytes (Tilley et al., 2001), splenic T cells (Nataraj et al., 2001), peripheral blood naive T cells, upregulated upon T cell activation (Boniface et al., 2009)
FP	No evidence for expression in T cells
BLT_1_	CD4+ and CD8+ effector T cells, particularly after activation (Tager et al., 2003; Islam et al., 2006), small fraction of peripheral blood T cells, including helper and cytotoxic T cells as well as NKT and γδ T cells (Yokomizo et al., 2001; Pettersson et al., 2003; Islam et al., 2006)
BLT_2_	CD4+ and CD8+ peripheral blood T cells, downregulated upon T cell activation (Yokomizo et al., 2001)
CysLTR_1_	Small fraction of peripheral blood T cells (Figueroa et al., 2001; Mita et al., 2001), activation induces higher expression (Prinz et al., 2005), as does IL-4 (Early et al., 2007). Significant amount in resting Th2 cells (Parmentier et al., 2012)
CysLTR_2_	Small fraction of peripheral blood T cells (Mita et al., 2001). IL-4 and IFN-γ induce expression (Early et al., 2007)

### PGI_2_/PC

Prostaglandin I_2_ was originally characterized as an inhibitor of platelet aggregation and a potent vasodilator (Boswell et al., 2011) and its analogs are used as treatments for pulmonary hypertension (Olschewski et al., 2004). Recently it has also been shown that this molecule has important roles in immune regulation (Boswell et al., 2011) and some studies suggest that treatment with PGI_2_ analogs may improve early graft viability in liver transplant patients, partly by reducing levels of inflammatory cytokines (Barthel et al., 2012).

While PGIS is expressed in some immune cells, in particular follicular dendritic cells (FDCs) (Lee et al., 2005; Boswell et al., 2011), there is no direct evidence for expression of this synthase in T cells. It has, however, been shown that lymphocytes are able to produce PGI_2_ through a transcellular mechanism when co-cultured with human vascular endothelial cells (HUVECs) (Merhi-Soussi et al., 2000) and that a similar mechanism appears to be operating between platelets and lymphocytes (Wu et al., 1987), although in neither of these cases were T cells specifically implicated.

The PGI_2_ receptor, IP, can be either G_s_ or G_q_-coupled, leading to either increases in intracellular cyclic AMP (cAMP) levels through G_s_-coupling, which can trigger cAMP-PKA signaling pathways or, through G_q_-coupling, to the initiation of other signaling cascades (Woodward et al., 2011). IP is expressed on T cells, in particular cells of the Th1 and Th2 lineages (Zhou et al., 2007). Signaling through the IP receptor on these cells leads to inhibited cytokine secretion – in particular, IFNγ production in Th1 cells is abrogated and Th2 cells express less IL-4, IL-10, and IL-13 after IP stimulation. These results are mirrored by studies in IP knockout mice, where IL-4 and IFNγ production by splenocytes, which includes some T cells, was significantly higher in sensitized IP KO mice than in WT mice (Takahashi et al., 2002). With the exception of IL-10, where other studies have also shown upregulation in response to IP signaling (Jaffar et al., 2002), these downregulated cytokines are proinflammatory, and PGI_2_ is generally considered to be an anti-inflammatory and immune suppressive prostaglandin.

This inhibitory effect of IP signaling on cytokine production from Th1 and Th2 cells appears to be mediated by a cAMP-PKA pathway, since the PKA inhibitor Rp-8-Br-cAMPS significantly reduces the IP-stimulation induced effects on cytokine production. Further, it is accompanied by a reduction in nuclear-factor kappa-light-chain-enhancer of activated B cells (NF-κB), a transcription factor known to enhance expression of IFNγ and IL-4 (Zhou et al., 2007). While signaling through the IP receptor has a direct negative regulatory effect on Th1 and Th2 function, it appears to promote differentiation into the Th17 lineage (Boswell et al., 2011; Truchetet et al., 2012). This effect is partially due to a reduction in IL-12 expression and/or an increase in IL-23 from dendritic cells (DCs) or monocytes, thus perturbing the IL-23 to IL-12 ratio and favoring Th17 cell differentiation (Boswell et al., 2011; Truchetet et al., 2012; Zhou et al., 2012). It appears that this pathway is IP-specific and proceeds through a PKA pathway (Truchetet et al., 2012). In addition, the favoring of the Th17 lineage during T cell differentiation upon IP-stimulation appears also to be due to inhibited secretion of IL-4 (Zhou et al., 2012), a cytokine known to promote Th2 and antagonize Th17 development (Boswell et al., 2011).

In addition to its role in regulating T cell differentiation, PGI_2_ also has an important role in mediating FDC-T cell interactions in the germinal centers. FDC-produced PGI_2_ has been shown both to inhibit T cell proliferation and to protect T cells from TCR-mediated activation-induced death (AICD) (Lee et al., 2005, 2008), thus improving the current understanding of why T cells don’t proliferate or undergo AICD in germinal centers.

### TXA_2_ and TXB_2_

Thromboxane A_2_ is a proinflammatory, short-lived (half-life *∼*30 s (Remuzzi et al., 1994)) prostanoid primarily produced in platelets, but also in activated monocytes, macrophages, and DCs (Narumiya, 2003) through the actions of thromboxane A_2_ synthase. There is limited evidence for TXAS expression in T cells and this synthase was found to be absent in thymic lymphocytes (Ushikubi et al., 1993). However, TXA_2_’s stable and inactive downstream metabolite, TXB_2_, is produced by helper T cells in response to isoproterenol stimulation (Genaro et al., 1992) and another study found very low levels of TXB_2_ produced from CD4+ cells (Kabashima et al., 2002), suggesting that TXAS could be present at low levels in certain T cells.

By signaling through the TP receptor, which is coupled to G_q_, TXA_2_ activates protein kinase C (PKC) and raises intracellular calcium levels (Narumiya, 2003; Woodward et al., 2011). TXA_2_ is best known for causing vasoconstriction and platelet aggregation and promotes fibrosis and scarring by regulating extracellular matrix protein levels (Thomas et al., 2003). The TP receptor is known to be highly expressed in immature thymocytes (CD4+CD8+ and CD4−CD8−) and to a lesser extent in CD4+CD8− and CD4−CD8+ thymocytes (Ushikubi et al., 1993; Kabashima et al., 2003). Splenic T cells also express lower amounts of the TP receptor. In line with this, immune regulatory functions for TXA_2_ have been proposed. Signaling through the TP receptor has been shown to cause apoptosis in immature thymocytes, in particular in CD4+CD8+ cells (Ushikubi et al., 1993), suggesting a potential role in T cell maturation. In other T cell populations, it has been suggested that signaling through the TP receptor could affect T cell proliferation (Kelly et al., 1979; Ceuppens et al., 1985), with a recent study showing that TXA_2_ signaling through the TP receptor inhibits anti-CD3 stimulated T cell proliferation (Thomas et al., 2003). Interestingly, proliferation in response to PMA and ionomycin, which produces a robust intracellular calcium response and bypasses the normal T cell activation mechanism, is not affected in TP-deficient cells (Thomas et al., 2003) or in cells treated with a TP agonist (Kabashima et al., 2003). These results indicate that TXA_2_ signaling through the TP receptor may play an important role in the initial activation of T cells by antigen-presenting cells (APCs), in particular DCs, but not in the later downstream intracellular signaling. TP signaling further attenuates DC-T cell interactions by promoting chemokinesis of naïve T cells and inhibiting DC-T cell adhesion, thus playing an important role in adaptive immunity (Kabashima et al., 2003).

Thromboxane A_2_ signaling has been implicated in anti-graft immune responses, with allografts eliciting higher levels of TXA_2_ than isografts (Gibbons et al., 1987). Mice deficient in the TP receptor have been shown to display weaker anti-allograft immune responses (Thomas et al., 2003) and blocking TXA_2_ synthesis pharmacologically has been shown to reduce alloreactive immune responses *in vitro* (Ruiz et al., 1992) and at least temporarily improve allograft survival and function *in vivo* by limiting cytotoxic T cell activity (Ruiz et al., 1989). On the other hand, in models of induced unresponsiveness to allografts by thymic injection of MHC allopeptides, TXA_2_ signaling abrogation through synthesis inhibition or receptor antagonists blocked the unresponsive state, suggesting that TXA_2_ signaling in the thymus is involved in mediating immune tolerance in this situation, possibly by leading to apoptosis of alloactivated T cells circulating through the thymus (Remuzzi et al., 1994). Together, these data suggests an important role for TXA_2_-TP signaling in T cells in the thymus, in particular in T cell maturation, activation by DCs and in anti-allograft immune responses.

### PGD_2_ and 15-deoxy-Δ^12,14^-PGJ_2_

PGD_2_ is produced by activated mast cells in response to allergen exposure and is thought to play an important role in mediating allergic inflammation by acting as a vasodilator, recruiter of eosinophils, basophils, and Th2 cells, modulator of Th2 production, and bronchoconstrictor (Pettipher et al., 2007). It also has important roles in regulating sleep, platelet aggregation, smooth muscle contraction, and reproduction (Saito et al., 2002; Woodward et al., 2011). Beyond mast cells, a few other cell types also produce PGD_2_ from PGH_2_ through one of the two types of PGD synthase, L-PGDS and H-PGDS (Joo and Sadikot, 2012). The former is not known to be expressed in T cells, while the latter is expressed in certain T cells under specific conditions. In particular, activated COX-2-expressing T cells have been shown to express H-PGDS and thereby produce PGD_2_ and likely the downstream PGD_2_ processing product 15-deoxy-Δ^12,14^-PGJ_2_ (15d-PGJ_2_) (Feldon et al., 2006). It appears that H-PGDS is particularly prevalent in activated Th2 and Tc2 cells but not Th1 cells (Tanaka et al., 2000; Herlong and Scott, 2006). As for the synthesis of 15d-PGJ_2_, no specific synthase has been described and few details are known about the dehydration steps leading to its formation from PGD_2_ (Scher and Pillinger, 2005).

PGD_2_ can signal through either the DP1 or DP2/CRTH2 receptor, while 15d-PGJ_2_ signals through the DP2 receptor (Harris et al., 2002a; Schuligoi et al., 2010). The DP1 receptor is G_s_-coupled and its activation leads to increases in intracellular cAMP and PKA activation and can also lead to increased intracellular calcium levels (Woodward et al., 2011). This receptor was shown to be expressed in certain malignant T cell lines (Harris and Phipps, 2002), but was not detected in normal peripheral blood T cells in this study (Harris and Phipps, 2002). However, other groups have found DP1 to be constitutively expressed in both Th1, Th2 and CD8+ cells (Tanaka et al., 2004) and to be present in CD3+ cells in the thymus and lymph nodes (Nantel et al., 2004). CRTH2 has little sequence homology with other prostanoid receptors, being more closely related to the *N*-formyl peptide receptor subfamily of receptors (Hirai et al., 2001). This receptor is G_i_-coupled, leading to increases in intracellular calcium and inhibition of cAMP formation in response to signaling (Hirai et al., 2001). It is thought to be mainly expressed in activated Th2 and Tc2 cells (Tsuda et al., 2001; Tanaka et al., 2004) and has also been detected in a subset of infiltrating T cells in patients suffering from polyposis, a severe form of rhinosinusitis (Nantel et al., 2004). Interestingly, when heterologously expressed, DP and CRTH2 can form heterodimers, where DP enhances the signaling by the CRTH2 receptor. In these heterodimers, when DP signaling is pharmacologically blocked, CRTH2 function is also inhibited, but not *vice versa* (Sedej et al., 2012). In addition to signaling through the cell surface receptors DP1 and CRTH2, 15d-PGJ_2_ and PGD_2_ can also bind the nuclear hormone receptor transcription factor peroxisome proliferator-activated receptor gamma (PPAR-γ) (Forman et al., 1995; Kliewer et al., 1995; Harris et al., 2002a; Feldon et al., 2006). By activating PPAR-γ, these prostanoids induce differentiation of fibroblasts into fat cells, and it has been shown that this can be pathophysiologically relevant. For instance, in the case of Graves’ disease, activated T cells infiltrate the eye orbit and by producing PGD_2_ and 15d-PGJ_2_, cause the differentiation of fibroblasts in the eye orbit to adipocytes, leading to disfiguration and sometimes blindness (Feldon et al., 2006).

Both PGD_2_ and 15d-PGJ_2_ affect cytokine production from T cells. In particular, 15-dPGJ2 is often thought of as an anti-inflammatory prostaglandin, in part due to its enhancement of PPARγ’s anti-inflammatory effects (Harris et al., 2002a; Scher and Pillinger, 2005). However, 15-dPGJ2 can also induce secretion of IL-8, a cytokine with chemotactic and angiogenic effects, from activated T cells, suggesting a proinflammatory role of this prostaglandin as well (Harris et al., 2002b). This effect is not PPAR-γ-dependent, but instead proceeds through a mitogen-activated protein kinase (MAPK) and NF-κB pathway, possibly by first binding an extracellular receptor such as CRTH2.

PGD_2_ has a well-established role in regulating cytokine secretion from Th2 cells. In particular, PGD_2_ produced in mast cells stimulates IL-4, IL-5, and IL-13 secretion from Th2 cells and this process is believed to be important in the pathophysiology of allergic inflammations (Xue et al., 2009a). It has been demonstrated that phosphoinositide 3-kinase (PI3K) and Ca_2+_/calcineurin/nuclear factor of activated T cells (NFAT) signaling pathways downstream of CRTH2 are both important in regulating PGD_2_-induced cytokine production (Xue et al., 2007) and that LTE_4_ enhances the PGD_2_-CRTH2-mediated secretion of cytokines from Th2 cells (Xue et al., 2012). Another study confirmed the effect of PGD_2_ receptor signaling on cytokine secretion and further noted that while signaling through CRTH2 increases secretion of IL-2, IL-4, IL-5, and IL-13 as well as the proinflammatory proteins CD11b and CD40L in Th2 cells, signaling through DP1 reduces the number of CD4+ and CD8+ cells expressing IFNγ and IL-2 (Tanaka et al., 2004). By thus promoting Th2 function and suppressing Th1 functions, PGD_2_ signaling may have an overall effect of promoting Th2 function, which could be relevant in allergic responses, where Th2 activity is elevated. PGD_2_ can have further inhibitory effects on cytokine secretion, for instance in invariant natural killer T (iNKT) cells, where PGD_2_ signals through DP1 and PKA to inhibit the production of IFNγ, but not IL-4, the other major cytokine produced in this cell type (Torres et al., 2008). Thus, PGD_2_ signaling also contributes to regulating the innate immune system.

While signaling through PGD_2_ receptors apparently has a role in driving Th2-type processes as described above, 15-dPGJ_2_ may have a role in resolving certain Th1-driven responses by inhibiting the proinflammatory NF-κB pathway (Trivedi et al., 2006). Also, 15-dPGJ_2_ is able to inhibit IL-2 production in T cells by promoting an interaction between PPARγ and NFAT, a crucial transcription factor for IL-2 production, which prevents NFAT from binding to the IL-2 promoter (Yang et al., 2000).

Aside from pro- and anti-inflammatory effects of PGD_2_ and 15-dPGJ_2_ mediated by cytokine secretion, these prostaglandins also affect T cell function by regulating proliferation and apoptosis. Both PGD_2_ and 15-dPGJ_2_ are capable of inducing apoptosis in T cells through a PPARγ-dependent mechanism (Harris and Phipps, 2001, 2002; Harris et al., 2002b). It has also been reported that 15-dPGJ_2_ and to a lesser extent PGD_2_ can induce apoptosis in Jurkat T cells through a non-PPARγ dependent mechanism involving activation of the mitochondrial apoptosis pathway (Nencioni et al., 2003). In other situations, PGD_2_ can also have anti-apoptotic effects. For instance, in the case of apoptosis induced by cytokine deprivation, PGD_2_ signaling through the CRTH2 receptor inhibits apoptosis in Th2 cells, suggesting that this pathway may hinder resolution of allergic inflammation (Xue et al., 2009b). In T lymphocytes, 15-dPGJ_2_ can also inhibit proliferation by acting as a PPARγ ligand (Clark et al., 2000; Yang et al., 2000; Harris and Phipps, 2001; Nencioni et al., 2003). However, only TCR-mediated and not IL-2 induced proliferation is affected by 15-dPGJ_2_ treatment (Clark et al., 2000).

Signaling through the PGD_2_ receptors also plays an important role in the chemotaxis of T cells. When PGD_2_ acts on the CRTH2 receptor on Th2 cells, this induces chemotactic migration of the Th2 cells (Hirai et al., 2001), probably through a PI3K pathway (Xue et al., 2007), providing a possible mechanism for recruitment of Th2 cells to sites of allergic inflammation, for instance in asthma (Luster and Tager, 2004). It has been demonstrated that blocking the CRTH2 receptor pharmacologically inhibits the trafficking of lymphocytes, including T cells, to the inflamed airways in a model of chronic obstructive pulmonary disease (COPD), presenting a possible new strategy for treating this disease (Stebbins et al., 2010). Further chemoattractive effects of PGD_2_ on T cells is the CRTH2-mediated recruitment of Th2 and Tc2 cells to the materno-fetal interface, where they are thought to increase in early pregnancy (Saito et al., 2002) and PGD_2_’s ability to promote transendothelial migration of memory T cells across blood vascular endothelial cells and lymphatic vascular endothelial cells (Ahmed et al., 2011).

### PGE_2_

PGE_2_ is the most abundant prostanoid found in the body and has important roles in reproduction, gastro-intestinal function, the immune system, cardiovascular function, and the central nervous system (Sreeramkumar et al., 2012). It is present in large amounts in many cancers, in particular colorectal and lung cancers, where it stimulates tumor growth by inhibiting apoptosis, inducing Tregs and promoting metastasis, cell invasion, and angiogenesis (Bergmann et al., 2007; Wang et al., 2007; Greenhough et al., 2009; Mandapathil et al., 2010; Brudvik et al., 2011; Nakanishi et al., 2011). PGE_2_ is produced from PGH_2_ through one of three different PGE_2_ synthases – cytosolic (cPGES) or microsomal (mPGES-1 and mPGES-2). While cPGES and mPGES-2 are constitutively expressed, mPGES-1 is inducible in response to mitogenic or proinflammatory stimuli and is often upregulated in concert with COX-2 (Scher and Pillinger, 2005). In terms of expression in T cells, little is known except that it has been demonstrated that adaptive Tregs express COX-2 and produce PGE_2_ upon differentiation (Mahic et al., 2006), implying that they must also express a PGES. This production of PGE_2_ from adaptive Tregs has implications both in cancer and chronic infectious diseases.

PGE_2_ can signal through any of its four receptors – EP1, EP2, EP3, EP4 – often with opposing effects (Breyer et al., 2001; Harris et al., 2002a; Woodward et al., 2011; Sreeramkumar et al., 2012). EP2 and EP4 receptors are G_s_-coupled and lead to increased intracellular cAMP levels and PKA signaling. The EP1 receptor is G_q_-coupled and results in increased intracellular calcium levels. In the case of the EP3 receptor, three main isoforms of this receptor exist – EP3 α, β, and γ – and they can signal through different G proteins, but it appears that the major pathway is through G_i_, which leads to decreased intracellular cAMP levels. Messenger RNA for all the different PGE_2_ receptors, with the exception of the EP3 α and β isoforms, is present in murine splenic T cells (Nataraj et al., 2001). In naive T cells isolated from peripheral blood, EP2 and EP4 receptors appear to be the most abundant and are upregulated in response to activation (Boniface et al., 2009).

Through its receptors, PGE_2_ controls T cell function in a variety of ways and a number of recent reviews have addressed this topic (Harris et al., 2002a; Brudvik and Tasken, 2012; Sreeramkumar et al., 2012). First, it appears to differentially regulate apoptosis in T cells depending on the subpopulation and condition of the cells. In particular, CD4+CD8+ thymocytes undergo apoptosis when stimulated by PGE_2_ *in vivo* (Mastino et al., 1992), but may also be protected against activation-induced cell death by this prostanoid (Goetzl et al., 1995). Similarly, while apoptosis is stimulated in resting mature T cells (Pica et al., 1996), activation-induced cell death is inhibited (Porter and Malek, 1999; Pace et al., 2007). PGE_2_ also has other known negative regulatory functions in T cells. It is known to influence the function of CD8+ cells through the inhibitory complex CD94/NKG2A (Zeddou et al., 2005) and the cytotoxicity of gamma delta T cells through a cAMP-PKA pathway (Martinet et al., 2010). An anti-proliferative effect is also well documented. Through the EP2 (Nataraj et al., 2001) and possibly the EP4 (Kabashima et al., 2002) receptor, PGE_2_ can inhibit T cell proliferation in CD4+ and CD8+ cells (Goodwin et al., 1977; Hendricks et al., 2000). It has also been shown that PGE_2_ inhibits the proliferation of double-negative Tregs (Lee et al., 2009). It appears that proliferation is inhibited in these cells by a negative regulatory effect of increased intracellular cAMP levels resulting from EP2 or EP4 stimulation on IL-2 synthesis and IL-2 receptor expression, resulting in diminished IL-2-stimulated proliferation responses (Farrar et al., 1987; Mary et al., 1987; Rincon et al., 1988; Anastassiou et al., 1992). Other possible mechanisms of inhibition of proliferation include downregulation of the transferrin receptor (Chouaib et al., 1985), inhibiting intracellular Ca_2+_ increase and inositol phosphate production in response to T cell activation (Chouaib et al., 1987; Lerner et al., 1988; Choudhry et al., 1999) and preventing K^+^ movements which would dampen signaling via G proteins (Bastin et al., 1990).

**Figure 2:**
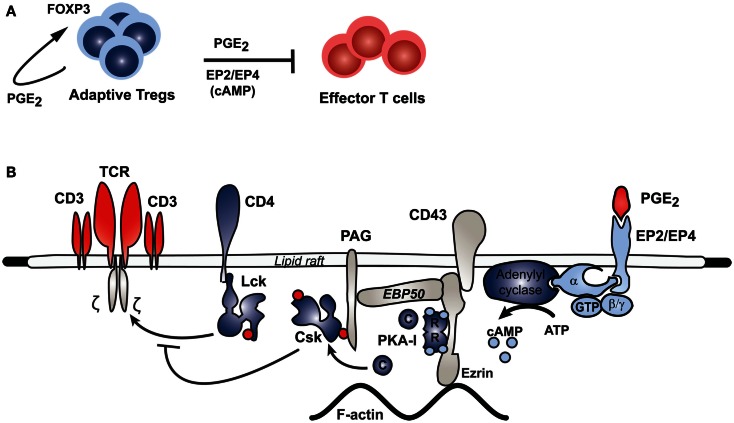
**Inhibitory pathway of PGE2 in effector T cells**. PGE_2_ mediates Treg inhibition of effector T cell function through a PKA-mediated pathway. **(A)** In response to continuous antigen exposure, for instance in cancer and HIV, adaptive regulatory T cells express COX-2 and produce PGE_2_, which stimulates FOXP3 expression in these cells. The Treg-derived PGE_2_ can signal through the EP2 and EP4 receptors on effector T cells to inhibit the function of these cells through the pathway shown in **(B)**. Binding of PGE_2_ to its receptors on effector T cells stimulates adenylyl cyclase activity, which increases intracellular cAMP levels and thus activates PKA. Aided by an Ezrin-EBP50-PAG scaffold, PKA phosphorylates Csk, which in turn phosphorylates Lck to inhibit its activity. Lck normally acts to promote TCR signaling; thus Lck inhibition through this PGE_2_-initiated pathway inhibits TCR signaling in effector T cells.

Recent studies have provided additional information about the intracellular signaling pathways initiated by PGE_2_ through which T cell function and proliferation is affected. In particular, a combined phosphoflow/phosphoproteomics approach allowed for the collection of detailed information about phosphorylation cascades initiated in response to different amounts of PGE_2_ stimulation in different T cell populations (Oberprieler et al., 2010). Furthermore, a pathway was described in effector T cells where signaling through EP2 or EP4, with its concomitant increase in cAMP levels, leads to PKA activation and, through an EBP50-Ezrin-PAG scaffolded process, phosphorylation of the C-terminal Src kinase (Csk). Phosphorylated Csk in turn inhibits Lck-mediated phosphorylation of the TCR complex, thus inhibiting TCR signaling and T cell proliferation and function (Vang et al., 2001; Ruppelt et al., 2007; Mosenden and Tasken, 2011) (see Figure 2 for schematic depiction of the PGE_2_-cAMP-PKA-Csk inhibitory pathway in T cells). This pathway is of particular relevance during inflammatory responses or cancer, where production of PGE_2_ is increased. It has been shown that disrupting this pathway in cells by molecular or genetic means prevents PGE_2_ – mediated inhibition of effector T cell function (Carlson et al., 2006; Ruppelt et al., 2007; Stokka et al., 2010). In mice, disrupting this pathway by overexpressing a PKA anchoring disruptor also leads to an increase in effector T cell function, as evidenced by increased signaling, enhanced IL-2 secretion, and reduced sensitivity to PGE_2_-mediated inhibition of T cell function. These mice also have improved resistance to murine AIDS, an immunodeficiency disease induced by the murine leukemia virus where the PKA-Csk pathway is hyperactivated (Mosenden et al., 2011). In mice with murine AIDS, this pathway can also be targeted with COX-2 inhibitors (Rahmouni et al., 2004). Interestingly, the PKA-Csk pathway is upregulated in several immunodeficiency diseases, as well as cancer, suggesting that targeting this pathway may be of therapeutic interest (Rahmouni et al., 2004; Brudvik and Tasken, 2012; Brudvik et al., 2012). In particular, targeting this pathway with COX-2 inhibitors in patients with HIV infection appears to give significant patient benefit in clinical intervention trials as evident from regulation of surrogate parameters such as CD38 and immune function parameters such as lymphoproliferation and T cell-dependent vaccine responses (Johansson et al., 2004; Kvale et al., 2006; Pettersen et al., 2011).

As described above, PGE_2_ can influence the production and secretion of IL-2 from T cells, but it also influences the production of many other cytokines and contributes to T cell differentiation. In particular, it has been proposed that PGE_2_ signaling promotes a Th2 cell fate (Betz and Fox, 1991). In line with this, PGE_2_ has been shown to downregulate expression of IFNγ in T cells (Aandahl et al., 2002), indicating less differentiation to a Th1 cell type, with the caveat that recent studies have shown that in the presence of strengthened TCR stimulation, the Th1 cell fate can actually be promoted by PGE_2_ (Yao et al., 2009). In contrast, Th2-derived cytokines including IL-4, IL-5, IL-10, and IL-13 are unaffected or upregulated in response to PGE_2_ signaling (Betz and Fox, 1991; Snijdewint et al., 1993; Demeure et al., 1997). Furthermore, the IL-12 receptor is downregulated on T cells in response to PGE_2_, further promoting a Th2 cell fate (Wu et al., 1998). PGE_2_ has also been proposed to play a role in the differentiation of Th17 and Tregs. There is some debate about the role of PGE_2_ in the differentiation and expansion of Th17 cells (Sakata et al., 2010b), with some studies finding an inhibitory role in mouse Th17 differentiation (Chen et al., 2009) and others finding a promoting role in human Th17 differentiation (Boniface et al., 2009). There seems to be general agreement that Th17 IL-23-mediated expansion is enhanced by PGE_2_, however (Chizzolini et al., 2008; Boniface et al., 2009; Napolitani et al., 2009). In Treg differentiation the majority of reports seem to suggest an enhancing effect (Baratelli et al., 2005; Sharma et al., 2005; Mahic et al., 2006; Bryn et al., 2008), although some have found PGE_2_ to have an inhibitory effect on this process (Chen et al., 2009). Due to its role in promoting Treg differentiation and inhibiting effector T cell function and proliferation, PGE_2_ has traditionally been considered an immunosuppressant, but with recent studies showing a possible enhancing effect of this eicosanoid on Th17 and Th1 differentiation, some have argued that the picture is more nuanced (Sakata et al., 2010b; Sreeramkumar et al., 2012).

### PGF_2α_

PGF_2α_ has important functions in reproduction, inflammation, cardiovascular function, and other (patho)physiological processes (Simmons et al., 2004; Basu, 2007, 2010; Woodward et al., 2011). This prostaglandin can be synthesized through a number of different pathways (Basu, 2010), but there appears to be no evidence for any PGF_2α_ synthesis in T cells. Evidence for a role of PGF_2α_ signaling in T cells is also very limited, although a recent study demonstrated a role for this prostaglandin in promoting Th17 differentiation during allergic lung inflammation (Li et al., 2011). In this study, the authors propose that PGF_2α_ together with PGI_2_ promotes differentiation of Th17 cells – proinflammatory cells and major contributors in allergic responses – from naïve CD4+ cells by signaling through their respective receptors in an autocrine fashion.

### Leukotrienes LTA_4_, LTB_4_, LTC_4_, LTD_4_, LTE_4_

The first step in leukotriene biosynthesis, conversion of arachidonic acid to the unstable epoxide intermediate LTA_4_, is catalyzed by 5-LOX, an enzyme shown to occur in human T cell lines as well as in purified peripheral blood T cells (Cook-Moreau et al., 2007). 5-LOX expression is found across a wide range of T cells, including naive and memory helper and cytotoxic T cells as well as TCR–γδ cells (Cook-Moreau et al., 2007). However, some have noted that T lymphocytes require exogenous arachidonic acid in order to synthesize leukotrienes (Cook-Moreau et al., 2007). This is interesting in light of the proposed transcellular eicosanoid biosynthesis mechanism, and it has also been shown that LTA_4_ can act as the transferred intermediate metabolite in some systems (Folco and Murphy, 2006; Sala et al., 2010). LTB_4_ synthesis, which proceeds through LTA_4_ hydrolase, and LTC_4_ synthesis, which proceeds through LTC_4_ synthase, both occur in Jurkat T cells upon CD2, CD3, and CD28 crosslinking (Cook-Moreau et al., 2007). In primary T cells, synthesis of LTB_4_ and LTC_4_ was only found to occur if cells were stimulated by CD3 crosslinking and supplied with exogenous arachidonic acid (Cook-Moreau et al., 2007). Depending on the stimulation protocol, others have also detected the production of LTB_4_ or the cysteinyl leukotrienes (LTC_4_, LTD_4_, LTE_4_) in various T cell lines and primary cells (Cifone et al., 1995; Los et al., 1995). It should be noted that LTD_4_ and LTE_4_ are typically generated extracellularly after export of LTC_4_ from the producing cell.

LTB_4_ can signal through either of its two receptors, the high affinity BLT_1_ receptor and the low affinity BLT_2_ receptor (Yokomizo et al., 1997, 2000), which couple to G_i_ or G_q_ to exert their function (Back et al., 2011). BLT_1_ is expressed on CD4+ and CD8+ effector T cells, particularly shortly after activation (Tager et al., 2003; Islam et al., 2006). In peripheral blood T cells in healthy humans, BLT_1_ is found on a small fraction of the population, including both helper and cytotoxic T cells as well as NKT and γδ T cells (Yokomizo et al., 2001; Pettersson et al., 2003; Islam et al., 2006), and can expand in response to acute inflammation. The BLT_2_ receptor is more ubiquitously expressed across tissues, with very high expression levels in the spleen (Yokomizo et al., 2000). One study found no evidence for BLT_2_ expression in naive CD4+ cells or Th0, Th1, or Th2 cells 7 days after activation (Tager et al., 2003), while others have shown it to be present on both CD4+ and CD8+ peripheral blood T cells, but downregulated in response to T cell activation (Yokomizo et al., 2001).

In T cells, LTB_4_ is primarily known for its role in chemotaxis, but it has also been shown to have other functions, for instance in differentiation and proliferation. In chemotaxis, LTB_4_ signals through the BLT_1_ receptor on CD4+ or CD8+ cells to mediate cell movement, which is of particular relevance during T cell recruitment to airways and lungs in asthma (Tager et al., 2003; Luster and Tager, 2004; Gelfand and Dakhanna, 2006), after lung transplants (Medoff et al., 2005) and in various inflammatory settings (Goodarzi et al., 2003; Ott et al., 2003). In addition, signaling through BLT_1_ appears to enable adhesion of T cells to epithelial cells (Tager et al., 2003), which is important for migration into tissues. In T cell differentiation, LTB_4_ has been shown to promote Th17 and inhibit Treg generation, which may be of relevance in autoimmune diseases such as rheumatoid arthritis (Chen et al., 2009). However, it should be noted that early reports from 1985 had suggested that LTB_4_ may have an immunoregulatory role by inducing so-called suppressor T cells (Yamaoka and Kolb, 1993; Morita et al., 1999) but this has not been revisited since the definition of Treg. Proliferation and cytokine production in T cells can also be affected by LTB_4_. In particular, treatment with a BLT_1_ antagonist was shown to inhibit cytokine (IL-2, IFN-γ, IL-4) secretion and proliferation of T cells in response to activation (Rolapleszczynski, 1985), while LTB_4_ stimulation enhanced IL-5 production (Gualde et al., 1985), suggesting that LTB_4_ promotes T cell activation.

For the cysteinyl leukotrienes LTC_4_, LTD_4_, and LTE_4_, two receptors have been discovered, CysLT_1_ and CysLT_2_ (Brink et al., 2003; Kanaoka and Boyce, 2004; Singh et al., 2010; Back et al., 2011; Laidlaw and Boyce, 2012). These receptors can bind all the cysteinyl leukotrienes, albeit with significantly higher affinity for LTC_4_ and LTD_4_ than LTE_4_ (Laidlaw and Boyce, 2012). Recently, further receptors involved in cysteinyl leukotriene signaling have been identified, in particular GPR17, which is a ligand-independent negative regulator of CysLT_1_, as well as the LTE_4_-specific P2Y_12_ (Austen et al., 2009; Maekawa et al., 2009; Paruchuri et al., 2009; Laidlaw and Boyce, 2012). While it is unclear whether these latter two receptors are expressed in T cells, the CysLT_1_ and CysLT_2_ receptors have been shown to be expressed in a small fraction of peripheral blood T cells (Figueroa et al., 2001; Mita et al., 2001). Activation of the T cells induces higher expression of the CysLT_1_ receptor (Prinz et al., 2005), as does certain mutations in the linker for activation of T cells (LAT) (Prinz et al., 2005). It also appears that expression of this receptor is significantly higher in resting Th2 cells than in Th1 cells or activated Th2 cells (Parmentier et al., 2012). Interestingly, both receptors can also be upregulated in response to inflammatory stimuli. In particular, IL-4 induces expression of both receptors on T cells, while IFN-γ specifically upregulates expression of the CysLT_2_ receptor (Early et al., 2007). Presumably, this upregulation has the effect of making T cells more responsive to cysteinyl leukotriene signaling in inflammatory environments.

There is some functional evidence for a role of cysteinyl leukotriene signaling in T cells. For one, these molecules appear to be important in Th2 cells where, as mentioned above, the CysLT_1_ receptor is present in significant amounts. It has been demonstrated that LTE_4_, through a montelukast-sensitive pathway, indicating CysLT_1_ involvement, enhances PGD_2_-mediated cytokine secretion in isolated Th2 cells (Xue et al., 2012). In line with this, another CysLT_1_ antagonist, pranlukast, inhibits production of Th2 cytokines, in particular IL-3, IL-4, GM-CSF and possibly IL-5, from peripheral blood mononuclear cells of patients with bronchial asthma (Tohda et al., 1999). Further roles in Th2 cells include a demonstrated effect of LTD_4_ on the induction of calcium signaling as well as chemotaxis in these cells, both processes being CysLT_1_-specific (Parmentier et al., 2012). Cysteinyl leukotriene signaling in Th2 cells may also be involved in disease. For instance, it has been suggested that cysteinyl leukotrienes may enhance GM-CSF stimulated Th2 functions in atopic asthmatic patients *in vivo* (Faith et al., 2008). There has also been a suggested role for the cysteinyl leukotrienes in T cell-mediated late airway responses to allergen challenge, since treatment with the CysLT_1_ antagonist pranlukast inhibits these responses (Hojo et al., 2000).

## Conclusion

Eicosanoids are an important class of lipid signaling mediators and have long been studied for their proinflammatory functions. In recent years, however, it has become evident that these molecules not only promote inflammation, but can occasionally also act as anti-inflammatory agents and have more complex and nuanced roles in the regulation of immune and inflammatory responses. Here, we have summarized the evidence for the expression of and signaling by some important eicosanoids, the AA-derived prostanoids and the leukotrienes, in T lymphocytes. These lipid mediators regulate a number of functions in T cells, including proliferation, apoptosis, cytokine secretion, differentiation, chemotaxis, and more. Through these processes, eicosanoids regulate a wide array of physiological processes, ranging from inflammatory processes such as asthma and allergies, to immune regulation and involvement in graft rejection, as well as diseases such as cancer and AIDS. There is significant interest in targeting some of these pathways for therapeutic gain and it is therefore crucial to develop a complete understanding of all the different physiological functions of these important signaling mediators.

## Conflict of Interest Statement

The authors declare that the research was conducted in the absence of any commercial or financial relationships that could be construed as a potential conflict of interest.
